# Supplementary material for: Prenatal versus Postnatal Initial Colonization of Healthy Neonates’ Colon Ecosystem by the Enterobacterium Escherichia coli
Source: Microbiol Spectr. 2021 Nov 24;9(3):e00379-21. doi: 10.1128/Spectrum.00379-21 (PMC8612161; doi:10.1128/Spectrum.00379-21)
Supplement: SUPPLEMENTAL FILE 1 — Supplemental material. Download SPECTRUM00379-21_Supp_1_seq9.pdf, PDF file, 0.1 MB [file spectrum00379-21_supp_1_seq9.pdf]

Supplementary Table 1: Strains and accession number of its 16S rRNA gene of *Escherichia coli* isolated from stool of healthy male newborn babies of normal birth vaginal delivery at first week of their life.

Strain	Accession No.	NCBI website link
E. coli TEM 105	MT928798	https://www.ncbi.nlm.nih.gov/nuccore/MT928798
E. coli TEM 107	MT928804	https://www.ncbi.nlm.nih.gov/nuccore/MT928804
E. coli TEM 104	MT928795	https://www.ncbi.nlm.nih.gov/nuccore/MT928795
E. coli TEM 109	MT928809	https://www.ncbi.nlm.nih.gov/nuccore/MT928809
E. coli TEM 111	MT912969	https://www.ncbi.nlm.nih.gov/nuccore/MT912969
E. coli TEM 162	MT928785	https://www.ncbi.nlm.nih.gov/nuccore/MT928785
E. coli TEM 116	MT912703	https://www.ncbi.nlm.nih.gov/nuccore/MT912703
E. coli TEM 119	MT912989	https://www.ncbi.nlm.nih.gov/nuccore/MT912989
E. coli TEM 127	MT920400	https://www.ncbi.nlm.nih.gov/nuccore/MT920400
E. coli TEM 141	MT921618	https://www.ncbi.nlm.nih.gov/nuccore/MT921618
E. coli TEM 156	MT928723	https://www.ncbi.nlm.nih.gov/nuccore/MT928723
E. coli TEM 122	MT918392	https://www.ncbi.nlm.nih.gov/nuccore/MT918392
E. coli TEM 132	MT921564	https://www.ncbi.nlm.nih.gov/nuccore/MT921564
E. coli TEM 144	MT928520	https://www.ncbi.nlm.nih.gov/nuccore/MT928520
E. coli TEM 160	MT928774	https://www.ncbi.nlm.nih.gov/nuccore/MT928774

Supplementary Table 2: Strains and accession number of its 16S rRNA gene of *Escherichia coli* isolated from stool of healthy female newborn babies of normal birth vaginal delivery at first week of their life.

**Strain**	**Accession No.**	**NCBI website link**
E. coli TEM 106	MT928802	https://www.ncbi.nlm.nih.gov/nuccore/MT928802
E. coli TEM 155	MT928721	https://www.ncbi.nlm.nih.gov/nuccore/MT928721
E. coli TEM 126	MT920363	https://www.ncbi.nlm.nih.gov/nuccore/MT920363
E. coli TEM 131	MT921541	https://www.ncbi.nlm.nih.gov/nuccore/MT921541
E. coli TEM 133	MT921582	https://www.ncbi.nlm.nih.gov/nuccore/MT921582
E. coli TEM 146	MT928518	https://www.ncbi.nlm.nih.gov/nuccore/MT928518
E. coli TEM 112	MT912572	https://www.ncbi.nlm.nih.gov/nuccore/MT912572
E. coli TEM 117	MT912717	https://www.ncbi.nlm.nih.gov/nuccore/MT912717
E. coli TEM 125	MT919313	https://www.ncbi.nlm.nih.gov/nuccore/MT919313
E. coli TEM 147	MT928531	https://www.ncbi.nlm.nih.gov/nuccore/MT928531
E. coli TEM 158	MT928725	https://www.ncbi.nlm.nih.gov/nuccore/MT928725
E. coli TEM 115	MT912692	https://www.ncbi.nlm.nih.gov/nuccore/MT912692
E. coli TEM 123	MT919071	https://www.ncbi.nlm.nih.gov/nuccore/MT919071
E. coli TEM 157	MT928724	https://www.ncbi.nlm.nih.gov/nuccore/MT928724
E. coli TEM 159	MT928752	https://www.ncbi.nlm.nih.gov/nuccore/MT928752

Supplementary Table 3: Strains and accession number of its 16S rRNA gene of *Escherichia coli* isolated from stool of healthy male newborn babies of cesarean section surgical delivery at first week of their life.

**Strain**	**Accession No.**	**NCBI website link**
E. coli TEM 134	MT921592	https://www.ncbi.nlm.nih.gov/nuccore/MT921592
E. coli TEM 143	MT928123	https://www.ncbi.nlm.nih.gov/nuccore/MT928123
E. coli TEM 164	MT928786	https://www.ncbi.nlm.nih.gov/nuccore/MT928786
E. coli TEM 101	MT102883	https://www.ncbi.nlm.nih.gov/nuccore/MT102883
E. coli TEM 118	MT912740	https://www.ncbi.nlm.nih.gov/nuccore/MT912740
E. coli TEM 130	MT921414	https://www.ncbi.nlm.nih.gov/nuccore/MT921414
E. coli TEM 135	MT921593	https://www.ncbi.nlm.nih.gov/nuccore/MT921593
E. coli TEM 153	MT928717	https://www.ncbi.nlm.nih.gov/nuccore/MT928717
E. coli TEM 128	MT921145	https://www.ncbi.nlm.nih.gov/nuccore/MT921145
E. coli TEM 129	MT921164	https://www.ncbi.nlm.nih.gov/nuccore/MT921164
E. coli TEM 148	MT928713	https://www.ncbi.nlm.nih.gov/nuccore/MT928713
E. coli TEM 154	MT928720	https://www.ncbi.nlm.nih.gov/nuccore/MT928720
E. coli TEM 161	MT928775	https://www.ncbi.nlm.nih.gov/nuccore/MT928775

Supplementary Table 4: Strains and accession number of its 16S rRNA gene of *Escherichia coli* isolated from stool of healthy female newborn babies of cesarean section surgical delivery at first week of their life.

**Strain**	**Accession No.**	**NCBI website link**
E. coli TEM 165	MT928787	https://www.ncbi.nlm.nih.gov/nuccore/MT928787
E. coli TEM 113	MT912573	https://www.ncbi.nlm.nih.gov/nuccore/MT912573
E. coli TEM 120	MT912993	https://www.ncbi.nlm.nih.gov/nuccore/MT912993
E. coli TEM 136	MT921598	https://www.ncbi.nlm.nih.gov/nuccore/MT921598
E. coli TEM 142	MT926457	https://www.ncbi.nlm.nih.gov/nuccore/MT926457
E. coli TEM 114	MT912687	https://www.ncbi.nlm.nih.gov/nuccore/MT912687
E. coli TEM 121	MT913593	https://www.ncbi.nlm.nih.gov/nuccore/MT913593
E. coli TEM 124	MT919307	https://www.ncbi.nlm.nih.gov/nuccore/MT919307
E. coli TEM 140	MT921613	https://www.ncbi.nlm.nih.gov/nuccore/MT921613
E. coli TEM 145	MT928530	https://www.ncbi.nlm.nih.gov/nuccore/MT928530
